# Photo-Responsive Carbon Capture over Metalloporphyrin-C_60_ Metal-Organic Frameworks via Charge-Transfer

**DOI:** 10.34133/research.0261

**Published:** 2023-10-24

**Authors:** Shi-Chao Qi, Zhen Sun, Zhi-Hui Yang, Yun-Jie Zhao, Jia-Xin Li, Xiao-Qin Liu, Lin-Bing Sun

**Affiliations:** State Key Laboratory of Materials-Oriented Chemical Engineering, Jiangsu National Synergetic Innovation Center for Advanced Materials (SICAM), College of Chemical Engineering, Nanjing Tech University, Nanjing 211816, China.

## Abstract

Great efforts have been devoted to the study of photo-responsive adsorption, but its current methodology largely depends on the well-defined photochromic units and their photo-driven molecular deformation. Here, a methodology to fabricate nondeforming photo-responsive sorbents is successfully exploited. With C_60_-fullerene doping in metalloporphyrin metal-organic frameworks (PCN-M, M = Fe, Co, or Ni) and intensively interacting with the metalloporphyrin sites, effective charge-transfer can be achieved over the metalloporphyrin-C_60_ architectures once excited by the light at 350 to 780 nm. The electron density distribution and the resultant adsorption activity are thus changed by excited states, which are also stable enough to meet the timescale of microscopic adsorption equilibrium. The charge-transfer over Co(II)-porphyrin-C_60_ is proved to be more efficient than the Fe(II)- and Ni(II)-porphyrin-C_60_ sites, as well as than all the metalloporphyrin sites, so the CO_2_ adsorption capacity (CAC; at 0 °C and 1 bar) over the C_60_-doped PCN-Co can be largely improved from 2.05 mmol g^−1^ in the darkness to 2.69 mmol g^−1^ with light, increased by 31%, in contrast to photo-irresponsive CAC over all C_60_-undoped PCN-M sorbents and only the photo-loss CAC over C_60_.

## Introduction

Traditional technologies of adsorption separation such as pressure swing adsorption, temperature swing adsorption, and vacuum swing adsorption always require large power quantities [1–4]. Stimuli-responsive adsorption separation is promising to provide high adsorption capability and selectivity but without additional power burden, such as guest-molecular and temperature-responsive adsorption [5–7]. In view of the great market prospect of solar energy, how to introduce optical factors in the adsorption separation are attractive, and thus, great efforts have been devoted to the study of photo-responsive or photo-stimuli sorbents [8–11]. Majority of current photo-responsive sorbents are designed to exploit the deformation of photochromic units [12–14], such as azobenzenes, diarylethenes, and spiropyrans, which are treated as the guest molecules to be integrated within the host materials like metal-organic frameworks (MOFs), covalent-organic frameworks (COFs), and mesoporous zeolites [15–17]. Once the photochromic units deformed owing to photo-stimulation, e.g., the trans-to-cis azobenzene and the open-to-close diarylethene ring with the ultraviolet (UV) irradiation, the steric effect or the polarity nearby the adsorption sites can be changed, resulting in the photo-modulated adsorption capability of the host material [18–22]. However, in most cases, the photo-responsive sorbents so prepared only exhibit the adsorption capability decrease with light, owing to the limitations of the deforming mechanism.

The photo-modulated adsorption capability does not perforce depending on the deformation of photochromic units when we realize that the specific adsorption capability exhibited by an adsorption site is ultimately attributed to its specific electron density distribution (EDD), which is invariable only at ground states, namely, Hohenberg–Kohn theorem [23]. Once the adsorption site is excited by photo-stimulation, the EDD can be evolved, and thus, the modulated adsorption capability can be expected even without molecular deformation [24,25]. Anyway, it is difficult to dramatically alter the EDD because the electron hole is largely overlapped for the local excitation with long lifetimes, whereas a charge-transfer (CT) excitation that can effectively alter the EDD is easily quenched, which cannot meet the timescale of microscopic adsorption equilibrium. The areas of artificial photosynthesis and organic photovoltaics related to light harvest and conversion afforded us experiences [26–28]. In these areas, the electron donor–acceptor systems were fabricated to attain long-lived CT states. In particular, fullerenes, as efficient π-electron acceptors due to highly delocalized π-electrons over the 3-dimensional π-sphere, can be coupled with porphyrins as the electron donors to form diverse dyads with efficient CT and energy-transfer processes [29]. For example, the CT state that resulted from the excitation of a triad, constructed by linking C_60_ and bis(3,4,5-trimethoxyphenyl)aniline to Al(III)-porphyrin, lies energetically 1.50 eV above the ground state [30].

**Fig. 1.**
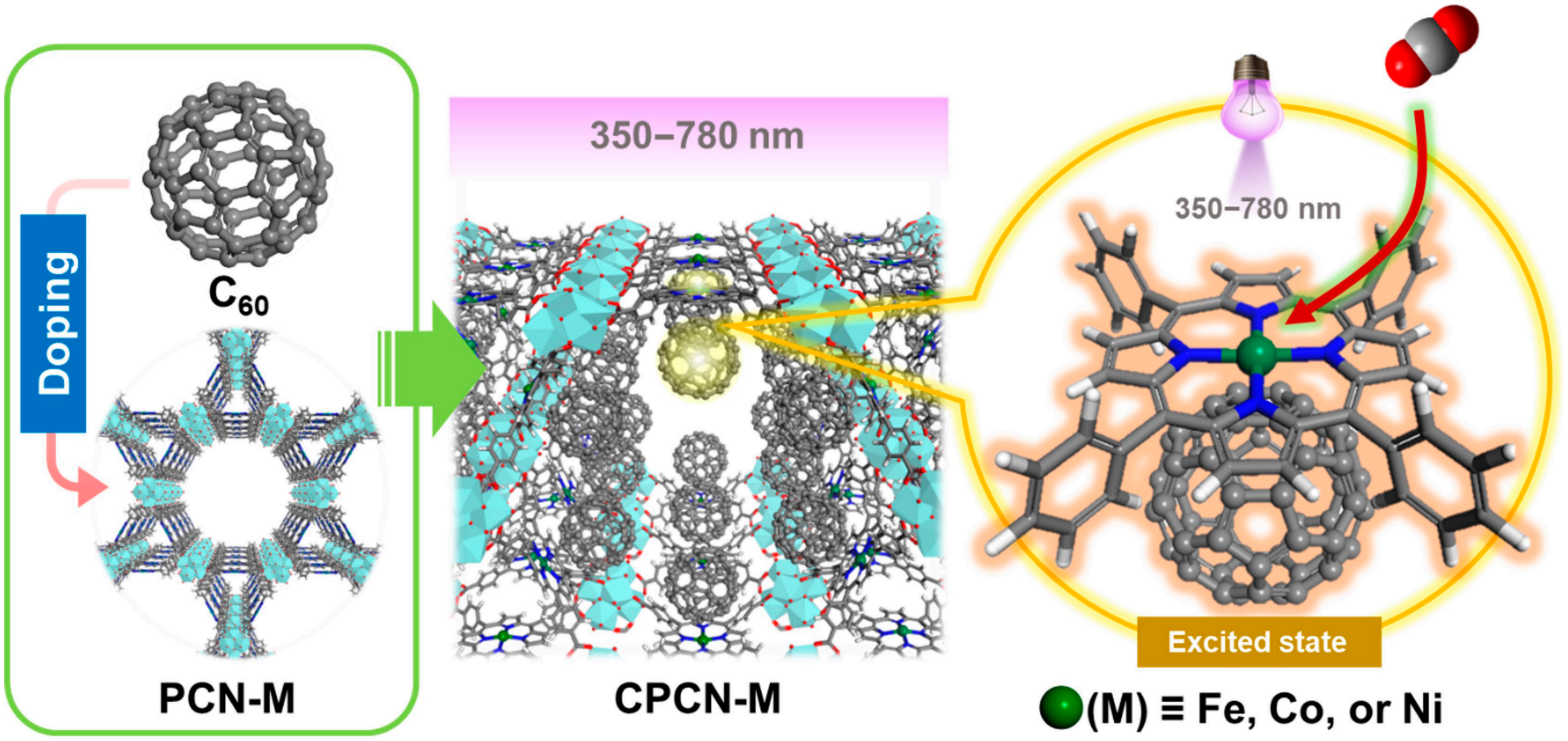
The scheme of the composite CPCN-M construction and the nonderforming photo-responsiveness to enhance CO_2_ adsorption capability over the metalloporphyrin-C_60_ site.

In this research, we expand the methodology of photoinduced CT state to the photo-responsive adsorption separation for the first time. Without photochromic units deforming, the methodology of nondeforming photo-responsiveness to efficiently modulate the EDD of adsorption sites is realized through photoinduced CT. We employed the metalloporphyrin MOF of PCN-222-M [PCN-M for short, and M = Fe(II), Co(II), or Ni(II)] and the fullerene of C_60_ to fabricate corresponding composite material, code-named CPCN-M, which are then used as the sorbents for the photoinduced selective adsorption of CO_2_ (Fig. 1). As a promising approach for carbon capture, adsorption separation has drawn much attention [31,32]. In particular, the direct air capture (DAC) as the new generation of carbon capture technology may have to mimic the biological photosynthetic carbon sequestration [33,34]. For example, microalgae cultures have promise as a CO_2_ sink for atmospheric carbon and as a sustainable source of food and chemical feedstocks [35]. Therefore, a sorbent that can exhibit enhanced CO_2_ adsorption capability in the sunlight sounds inspiring. Here, all the C_60_-undoped PCN-Ms do not exhibit noticeable changes of CO_2_ adsorption capability with UV-visible (UV-Vis) irradiation; meanwhile, the CO_2_ uptake capacity over C_60_ only decreases with the UV-Vis irradiation. In contrast, their composite sorbents CPCN-Ms exhibit marked photo-responsiveness in terms of CO_2_ adsorption capability. For instance, the CO_2_ adsorption capacity (0 °C, 1 bar) of CPCN-Co is elevated from 2.05 mmol g^−1^ in the darkness to 2.69 mmol g^−1^ with the UV-Vis irradiation, increased by 31%. Moreover, even though the porphyrin-coordinated metals in CPCN-Ms, i.e., Fe, Co, and Ni, only differ by an electron, we prove that their CT modes and resultant macroscopical adsorption performances with the UV-Vis are totally different from each other.

## Results

### Materials characteristics

**Fig. 2.**
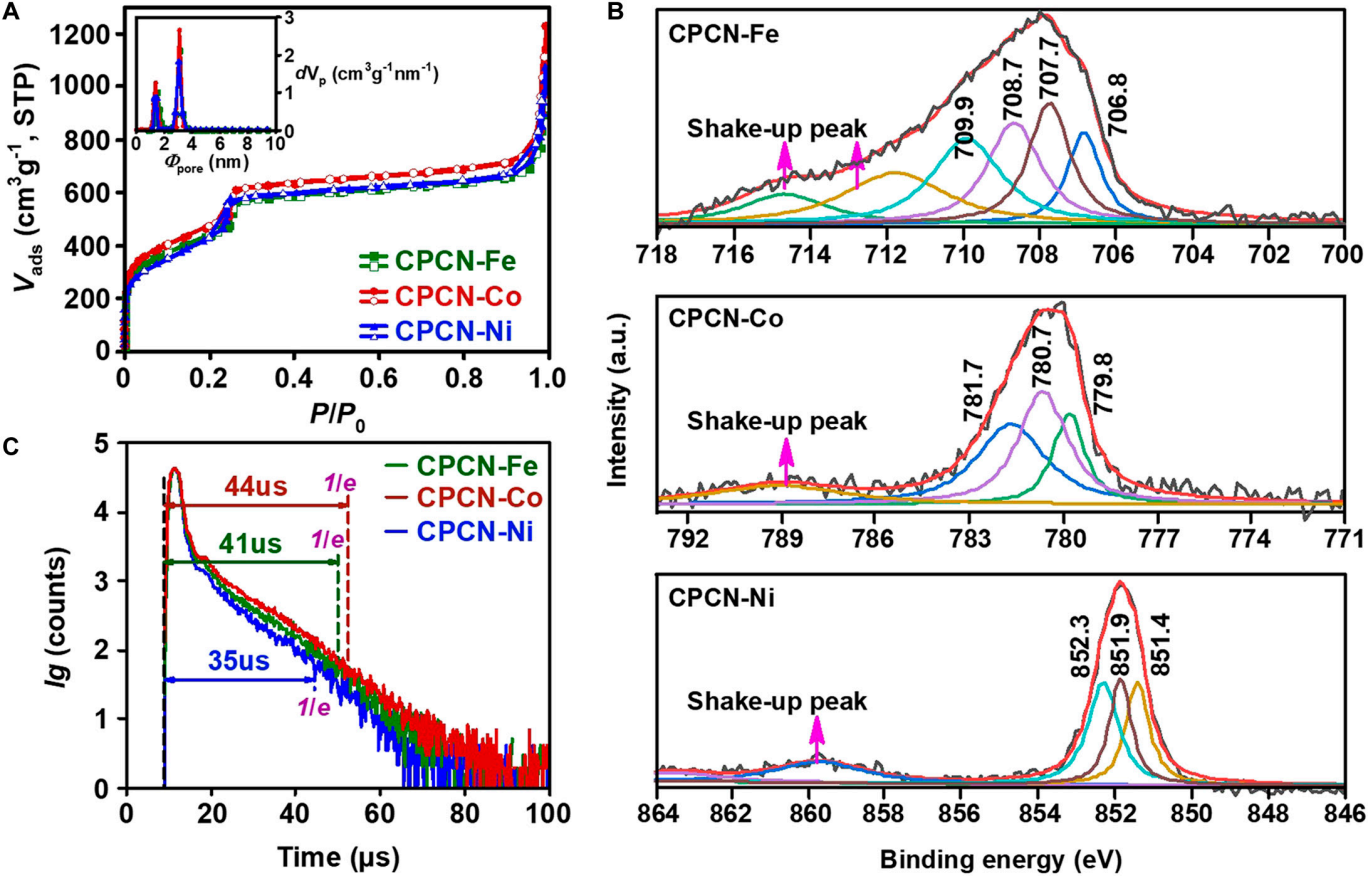
Textural, optoelectronic, and phosphorescent properties of CPCN-Ms. (A) N_2_ adsorption–desorption isotherms and pore size distribution (inset). (B) XPS deconvolution for the 2*p*3/2 band of the coordinated Fe, Co, and Ni. (C) Phosphor decay profiles at 750 nm, in which 1/*e* indicates the effective phosphorescence lifetime.

Unless otherwise noted, the mass ratio between C_60_ and PCN-M for CPCN-M is 1:8, which exhibits better adsorption capability and more obvious photo-responsiveness than those with other mass ratios, as discussed in the following section. The crystalline grains of as-prepared CPCN-Ms are neat and uniform, and introducing C_60_ does not influence the successful crystallization (Fig. S1). It can be further confirmed through x-ray powder diffraction (XRPD). The XRPD patterns of CPCN-Ms, which are also not changed by the type of the porphyrin-coordinated metal, are consistent with that of PCN-M reported (Fig. S2) [36–38]. The successful preparation of CPCN-Ms can be proved by the Fourier transform infrared (FTIR) spectra. The characteristic band of C_60_ at 1180 cm^−1^ is retained for the FTIR spectra of CPCN-Ms, in which the characteristic bands located at 1006 cm^−1^ indicate that the presence of porphin-*N*-metal bonds can also be seen (Fig. S3). In the high-resolution electron microscopy (HREM) images (Fig. S4), the structured C_60_ aggregation attached to the MOF grain crystals can be seen occasionally, and a considerable number of C_60_ molecules ought to be monodispersed among the crystal lattices of the MOFs, manifested as bright spots with the size of 0.7 nm at the accelerating voltage of 200 kV [39,40]. Moreover, owing to the doped C_60_ molecules, the observed lattice distance (ca. 2.6 nm) of CPCN-M is stretched compared to the theoretical value (ca. 2.1 nm) of the PCN-M [200] plane, and the lattice is somewhat distorted. The crystal forms of different CPCN-Ms are similar to each other, as well as their textural properties. As shown in Fig. 2A, all the N_2_ adsorption–desorption isotherms of CPCN-Ms feature typical IV type and exhibit a steep increase at low *P*/*P*_0_ and an increase at *P*/*P*_0_ = 0.25, suggesting microporosity and mesoporosity. The Brunauer–Emmett–Teller (BET) specific surface areas (*S*_BET_) of CPCN-Fe, CPCN-Co, and CPCN-Ni are 2100, 2160, and 2130 m^2^ g^−1^, respectively (Table S1). Note that *S*_BET_ of C_60_ is lower than 10 m^2^ g^−1^ (Fig. S5), so the high *S*_BET_ values of CPCN-Ms are mainly from the host PCN-Ms. There are 2 types of pores for all CPCN-Ms, with sizes of 1.4 and 3.0 nm (Fig. 2A, inset), assigned to their triangular micro- and hexagonal meso-channels, respectively. The undifferentiated textural properties of CPCN-Ms ensure that their adsorption performances only depend on the intrinsic activity of the adsorption site, i.e., the metalloporphyrin ring, either at the ground state or at excited state.

Although the dispersed C_60_ ought to interact with the host PCN-M via van der Waals’ forces [41], it can be proved that the interaction is intense. According to the thermogravimetric (TG) profiles, the weight loss at the temperature range higher than 650 °C can be observed for all CPCN-M samples, implying the intense interaction between C_60_ and PCN-M (Fig. S6). Moreover, the residual weights shown in the TG profiles conform to the theoretical ash contents of CPCN-Ms. The H-nuclear magnetic resonance (H-NMR) provides more convincing evidence. Taking the H-NMR of PCN-Co and CPCN-Co, for example (Fig. S7), owing to the shielding effect caused by C_60_, the chemical shift *δ* of the porphin ring-*H* moves to higher magnetic field compared to that of PCN-Co, and correspondingly, the *δ* of both the proximal and the distal phenyl-*H* moves to lower magnetic field due to the deshielding effect. The intense interaction between C_60_ and the host PCN-M further influences the presentational valences of the porphyrin-coordinated metals. In Fig. 2B, obvious shake-up peaks can be observed in all x-ray photoelectron spectroscopy (XPS) spectra of CPCN-Ms, indicating that the experimentally introduced metals exist in the status of high valence, i.e., Fe(II), Co(II), or Ni(II), and the bivalence exhibits as the deconvolved peaks under their 2*p*3/2 bands correspondingly, such as those located at 708.7 and 709.9 eV for Fe(II), 781.7 eV for Co(II), and 852.3 eV for Ni(II), and other deconvolved peaks located at lower binding energies indicate the lower presentational valences for all CPCN-Ms. For example, the peaks located at 707.7 and 706.8 eV for CPCN-Fe should be caused by the porphyrin ring with large conjugate orbitals donating electrons and C_60_-porphyrin ring cooperatively donating electrons to the coordinated Fe(II), respectively. The C_60_-porphyrin ring cooperatively donating electrons is so intensive that the coordinated Fe(II) exhibits a tendency to be reduced to Fe(0) [42]. Such intense interaction between C_60_ and the porphyrin-coordinated metals is crucial for the photoinduced adsorption because the core metal is the decisive factor for CT and the formation of excited states.

### Photoinduced adsorption

We have previously proved that although the metalloporphyrin and their derivatives can be excited to diverse orders with the UV or UV-Vis irradiation, de-excitation processes such as internal conversion, vibrational relaxation, and intersystem crossing must be rapid, and ultimately, the most stable low-order excited states will alter the EDD dominantly [24,25]. Without molecular deformation, the excitation-altered EDD substantially different from the EDD at the ground state must take an effective CT as a prerequisite, and the lifetime of the low-order excited state must be long enough to meet the timescale of molecular adsorption equilibrium (~10^−6^ s) [43,44]. With the 420-nm irradiation, the phosphorescent radiation related to the low-order excited states can be detected for all CPCN-Ms (Fig. S8). The corresponding phosphor decay profiles demonstrate that the effective lifetimes of the excited states for CPCN-Fe, CPCN-Co, and CPCN-Ni have reached 41, 44, and 35 μs, respectively (Fig. 2C). The lifetimes are durable enough to meet the molecular adsorption equilibrium. In contrast, although the phosphorescent radiation of C_60_ can also be detected, its effective lifetime is only 6 μs (Fig. S9). Moreover, in view of the poor textural property of C_60_ as mentioned above, it is safe to say that the isolated C_60_ aggregation, if any, would not perturb the investigation for the adsorption capabilities of the composite sorbents either at the ground state or at the excited state.

**Fig. 3.**
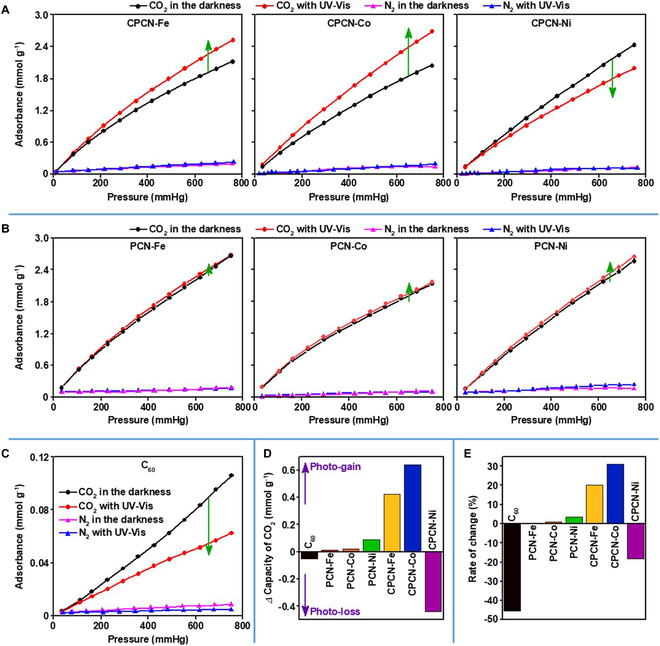
The static adsorption isotherms of CO_2_ and N_2_ tested with UV-Vis irradiation and in the darkness over CPCN-Ms and PCN-Ms at 0 °C. (A) Static adsorption isotherms of CO_2_ and N_2_ over CPCN-Ms, in which the green arrows indicate the variation trend of the UV-Vis CO_2_ adsorption isotherm with respect to that in the darkness. (B) Static adsorption isotherms of CO_2_ and N_2_ over PCN-Ms. (C) Static adsorption isotherms of CO_2_ and N_2_ over C_60_. (D) Difference of the CO_2_ uptake capacity at 1 bar with UV-Vis subtracting that in the darkness. (E) Rate of change for the photo-responsive CO_2_ adsorption capacity at 1 bar.

Owing to the intrinsic high *S*_BET_ value and microporosity, CPCN-Ms exhibit selective adsorption of CO_2_ at ground states. As shown in Fig. 3A and Table S1, the adsorption capacities of CPCN-Fe, CPCN-Co, and CPCN-Ni in the darkness for CO_2_ reach 2.08, 2.05, and 2.43 mmol g^−1^ at 0 °C and 1 bar, whereas those for N_2_ are only 0.20, 0.14, and 0.13 mmol g^−1^, respectively. The initial selectivity of CO_2_ toward N_2_ calculated with ideal adsorption solution theory can reach 124, 132, and 144, respectively (Fig. S10). As for the photoinduced adsorption experiments, the UV-Vis light with the wavelength of 350 to 780 nm was employed to sufficiently excite the sorbents because CPCN-Ms exhibited strong absorption at a wide UV-Vis range (Fig. S11). With the UV-Vis light, the CO_2_ adsorption capacities of CPCN-Fe and CPCN-Co are elevated to 2.50 and 2.69 mmol g^−1^ at 0 °C and 1 bar, increased by 20% and 31%, respectively, while that of CPCN-Ni decreases to 1.99 mmol g^−1^ at 0 °C and 1 bar, with the loss rate of 18% instead (Fig. 3A, D, and E and Table S1). Compared to the CO_2_ adsorption markedly changed by the UV-Vis irradiation, the UV-Vis influence on the N_2_ adsorption over CPCN-Ms is imperceptible. The initial selectivity of CO_2_ toward N_2_ is thus changed to be 140, 164, and 108 for CPCN-Fe, CPCN-Co, and CPCN-Ni, respectively. In other words, the photo-gained adsorption capability of CO_2_ can be achieved only over CPCN-Fe and CPCN-Co, and CPCN-Co exhibits more significant photo-responsiveness than CPCN-Fe.

As for the undoped materials, i.e., C_60_, PCN-Fe, PCN-Co, and PCN-Ni, none of them exhibits obvious photo-responsiveness in terms of selective CO_2_ adsorption (Fig. 3B and C). It is no wonder that the CO_2_ uptake capacity of C_60_ at 0 °C and 1 bar is only 0.11 mmol g^−1^ in the darkness, and then decreases to 0.06 mmol g^−1^ with the UV-Vis irradiation, because its textual property is so poor as mentioned above, and as discussed below, the decrease of adsorption activity over C_60_ at the excited state must cause the CO_2_ desorption. In contrast to the obvious photo-desorption of CO_2_ over C_60_, a noteworthy fact is that the CO_2_ adsorption isotherms with the UV-Vis are almost coincided with those in the darkness for all PCN-Ms. This means that the photo-responsiveness of pristine PCN-Ms, if any, is too weak to dramatically alter the CO_2_ adsorption capability. These control experiments further verify the necessity of doping C_60_ in PCN-Ms to generate effective photo-responsiveness. Anyway, the doping amount of C_60_ should be optimized to balance the CO_2_ adsorption capacity and the photo-responsiveness, and thus, the C_60_/PCN-M mass ratio of 1:8 is proved to be appropriate (Fig. S12). The photo-responsiveness exhibited by CPCN-Ms ought to be strong enough, which is observable even at 25 °C (Fig. S13). Among the CPCN-M sorbents, the photoinduced CO_2_ adsorption performance of CPCN-Co is competitive with other representative photo-responsive CO_2_ sorbents during the past decade, especially when we consider that majority of the reported photo-responsive sorbents had to depend on deforming units, and the UV-Vis irradiation only caused the desorption over those sorbents (Table S2) [19,20,45–52]. Moreover, as the representative CPCN-M sorbent, CPCN-Co shows ideal ex situ reversibility, of which CO_2_ adsorption capacity both at the ground state and with the UV-Vis irradiation can be well maintained even after five cycles (Fig. S14).

### Mechanisms of photo-responsiveness

As mentioned above, owing to the C_60_ doping in the host PCN-Ms, all CPCN-Ms exhibit markedly nondeforming photo-responsiveness in terms of CO_2_ adsorption capability, while the photo-responsiveness of CPCN-Fe and CPCN-Co exhibits as photo-gained CO_2_ adsorption capability, and that of CPCN-Ni presents as the photo-loss on CO_2_ uptake capacity. In contrast, none of the host PCN-Ms exhibits obvious photo-modulated effects on CO_2_ adsorption compared to that without UV-Vis light. The underlying mechanisms are revealed through the calculations based on density functional theory (DFT) and time-dependent DFT (TDDFT). Here, tetraphenylporphyrin-M (TPP-M, M = Fe, Co, or Ni) is employed to simulate the corresponding adsorption site of PCN-M [24], and the C_60_-TPP-M architecture (CTPP-M) is used to simulate that of CPCN-M, referring to Guldi’s literature [41], of which C_60_ is located at the *c* axis of the porphin ring.

**Fig. 4.**
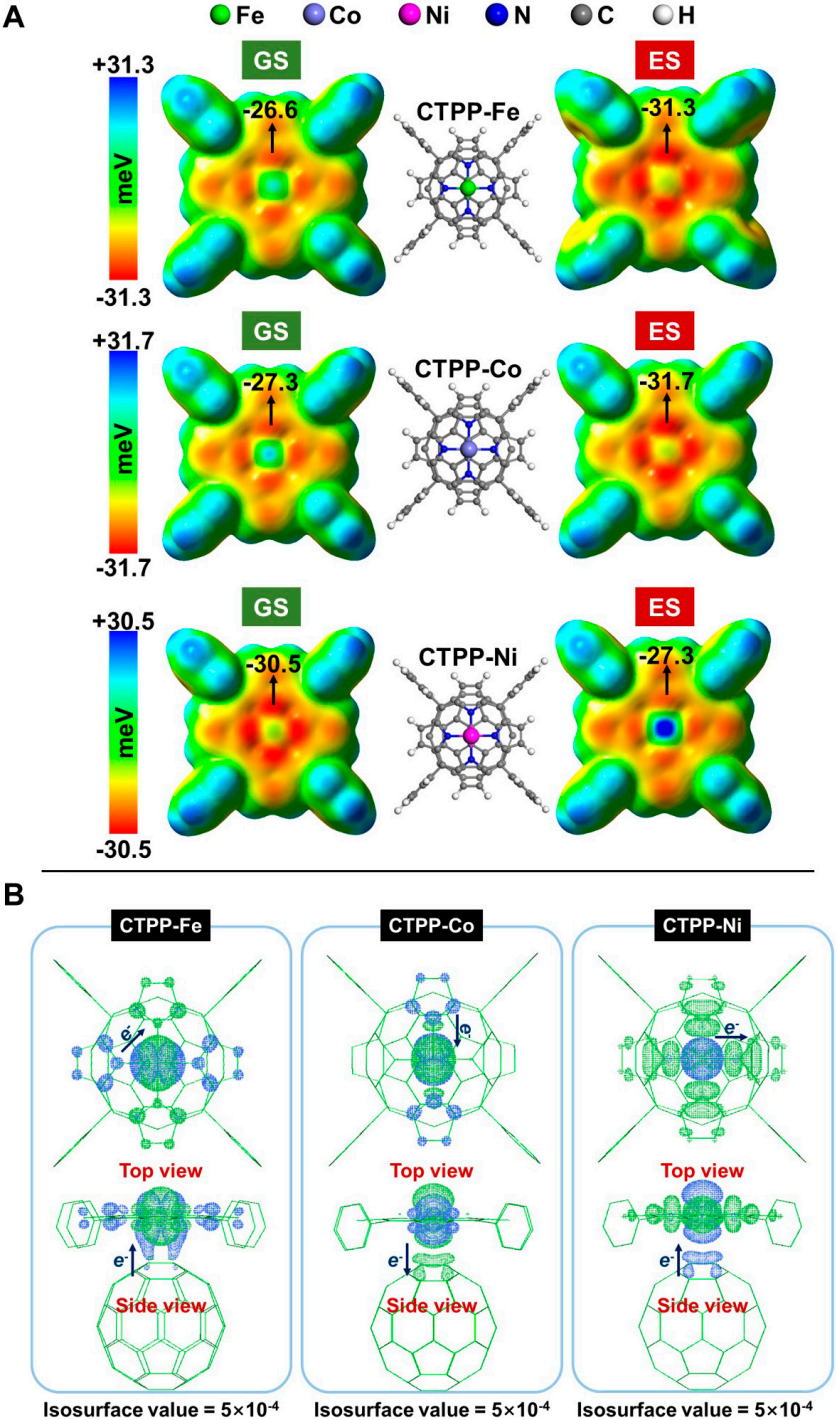
The effect differences between the EDD at the excited state (ES) and that at the ground state (GS) for CTPP-M. (A) Molecular surface ESPs with the density isovalue = 1 × 10^−3^ at the ground and excited states. (B) The electron-hole distribution at the excited state (green area, electron distribution; blue area, hole distribution).

As for the non-bonded interaction like adsorption, we have proved that the nature of adsorption site can be indicated by the molecular surface electrostatic potential (ESP) [21,24,53]. The negative ESP area can be seen over TPP-Ms even at the ground state, which is mainly contributed by the conjugated electrons of porphin-*N* atoms, and the negative ESP interacts with the electron-deficient π_3_^4^ orbitals of the CO_2_ molecule as strong van der Waals’ force to capture it (Fig. S15). In contrast, the electron-full π_2_^4^ and σ_2_^2^ orbitals of N_2_ molecule are much less prone to be induced by the negative ESP, which results in the high selectivity of CO_2_ toward N_2_ over the adsorption sites. Once TPP-M is excited, the original EDD at the ground state can be altered such that the maximal negative ESP is enhanced, which can induce and capture the CO_2_ molecule more intensely. However, the enhancement of the negative ESP seems to be limited. For example, the maximal negative ESP over the TPP-Co is only changed from −30.0 meV at the ground state to −30.8 meV at the excited state. Such enhancement level might be just enough to offset the repulsion among the CO_2_ molecules that are more gathered nearby the adsorption site due to the induction force enhanced with the negative ESP. Therefore, there is no significant difference between the CO_2_ adsorption isotherms in the darkness and those with the UV-Vis light. As for CTPP-Ms, the excited states alter the EDD and the resultant ESP over the porphin ring powerfully due to the presence of C_60_. As Fig. 4A exhibits, the maximal negative ESP values of CTPP-Fe and CTPP-Co are enhanced from −26.6 meV at the ground state to −31.3 meV at the excited state, and from −27.3 to −31.7 meV, respectively, whereas that of CTPP-Ni is weakened from −30.5 to −27.3 meV instead. Such obvious changes of ESP over the adsorption sites are enough to produce remarkable results on a macro level, and thus, the CO_2_ adsorption isotherms over all CPCN-Ms can be markedly changed due to the UV-Vis irradiation. What is more, the different ESP changes over CTPP-Ms can well explain the experimental results of photo-gain for CPCN-Fe and CPCN-Co, but photo-loss for CPCN-Ni in terms of CO_2_ adsorption capability.

The very different response modes of abovementioned adsorption sites to the UV-Vis excitation should be ascribed to the core metals and whether there is C_60_. With the electron-hole distribution analysis for all the adsorption sites at excited states [54,55], both electrons and holes are mainly contributed by the core metals (Table S3). The DFT calculations prove that all TPP-Ms and CTPP-Ms are inner-orbital configurations, so the electronic configurations of the coordinated metals should be Fe-*d*[22110], Co-*d*[22210], and Ni-*d*[22220]. Because there are 2 and 1 unpaired *d*-electrons for Fe and Co, respectively, they are prone to accept the conjugated electrons from the porphin ring once excited. In contrast, Ni is prone to donate its electrons at the excited state. This is why the core metal can participate in the EDD alteration via excitation. As for TPP-Ms, the rearranged electrons are enriched over the core metals, resulting in an enhanced negative ESP (Fig. S16). However, both electrons and holes at excited states are mainly contributed by the core metals, and the electron hole is largely overlapped for TPP-Ms; in other words, the CT state over TPP-Ms is inapparent, so the enhancement of the negative ESP over TPP-Ms is limited.

As for CTPP-Ms, the *d* orbitals of the core metal are coupled with the 3-dimensional π-orbitals of C_60_ at the *c*-axis direction, which makes it possible to realize the CT state. As shown in Fig. 4B, the holes at excited states contributed by Fe and Ni are coupled with those by C_60_, respectively, as well as the electrons at excited states contributed by Co. Although both the electrons and holes of the excited CTPP-Ms are still mainly contributed by the *d*-orbitals of the core metals, the symmetry of these areas is largely broken both at the *c*-axis direction and on the porphin ring, realizing the CT states. As a result, the variation of dipole moment *μ* with respect to the ground state for CTPP-M is much larger than that for the corresponding TPP-M due to CT at the *c*-axis direction, and the electron/hole delocalization indexes of the excited CTPP-M are all less than those of the excited TPP-M, indicating more delocalized electron/hole of CTPP-M than that of TPP-M due to CT on the porphin ring (Table S3). The excitation resultant CT states over CTPP-Ms are different from each other due to the different electron configurations of the core metals. The coordinated Fe ion of CTPP-Fe with 2 unpaired *d*-electrons can accept the electrons not only from the porphin ring but also from C_60_ at the excited state; meanwhile, the electrons from the porphin ring cannot be completely donated to the core Fe owing to the mismatched symmetry between orbitals, so the final result is that CT occurs not only between TPP-Fe and C_60_ but also between different areas on the porphin ring (Fig. 4B). The Co ion in CTPP-Co with one unpaired *d*-electron only causes electron donating from the porphin ring, and C_60_ also provides π-orbitals to jointly accept the electrons such that the electrons enriched at the *c*-axis direction are beneficial to the enhancement of negative ESP. The fully paired electrons of the Ni ion in CTPP-Ni once excited must be donated, and the hole coupled with C_60_ decentralizes the electrons on the porphin ring severely, so the negative ESP nearby the core Ni is weakened at the excited state. In addition, C_60_ itself cannot act as the CO_2_ adsorption site, in view of its nearly neutral ESP at either the ground or excited state (Fig. S17), of which electron hole is almost completely overlapped in all directions on the C_60_ sphere (Fig. S18). In fact, the maximal negative ESP over C_60_ is even increased from −2.0 meV at the ground state to −0.2 meV at the excited state (Fig. S17), clearly indicating the decrease of its adsorption activity toward CO_2_ at the excited state and explaining its reduced CO_2_ adsorption capacity with the UV-Vis irradiation. Moreover, as a carbonaceous material, C_60_ may have the heat effect caused by the UV-Vis irradiation, with which the CO_2_ capacity is reduced.

## Discussion

The nondeforming photo-responsiveness of metalloporphyrin MOFs, i.e., PCN-222-Fe(II), PCN-222-Co(II), and PCN-222-Ni(II), is exploited in this study, with doping C_60_ in the host MOFs to construct the composite sorbents. In contrast to the current methodology to fabricate photo-responsive sorbents depending on the mechanical deformation of photochromic units, the nondeforming photo-responsiveness here is based on markedly changing the EDD over the metalloporphyrin adsorption sites via effective CT caused by excited states. Owing to the intense interaction of metalloporphyrin-C_60_ and their orbitals coupling, an effective CT path is built among the porphin ring, the coordinated metal, and C_60_. The unpaired *d*-electrons make the porphin ring coordinated Fe(II) and Co(II) the electron-acceptors, over which the excited electrons can be enriched to enhance their adsorption activity, whereas the porphin ring coordinated Ni(II) with fully paired *d*-electrons acts as the electron-donor instead such that its excited electrons are decentralized to reduce the adsorption activity severely. In addition to the effective CT that modulates the EDD of adsorption sites, the low-order excited states of the composite sorbents possess long lifetimes, which meet the timescale of molecular adsorption equilibrium. As a result, the CO_2_ adsorption capabilities of all the composite sorbents are obviously changed, and especially that of the Co(II)-coordinated one is significantly increased, with the UV-Vis irradiation.

## Materials and Methods

### Materials synthesis

All involved chemicals were commercially purchased and used as received. The materials are prepared according to the reported recipes with slight modifications [36–38]. To prepare the ligand of TCPP-M, the solution of 5,10,15,20-tetrakis(4-methoxycarbonylphenyl)porphyrin (TPPCOOMe, 1.0 mmol; purity > 95%; Yanshen, Jilin) and superfluous corresponding metal salt (FeCl_2_, CoCl_2_, or NiCl_2_) in 100 ml of dimethyl formamide (DMF) was refluxed for 8 h. With H_2_O added in the solution, the generated precipitate was filtered and then washed with H_2_O sufficiently. The solid intermediate was dissolved in CHCl_3_ and then sufficiently acidized with 1 M HCl. Washed with H_2_O and dried with MgSO_4_, the organic phase was evaporated to obtain the TCPP-M (M = Fe, Co, or Ni).

To prepare CPCN-M, C_60_-fullerene (6 mg; purity > 99.9%; Macklin, Shanghai) was ultrasonically dissolved in toluene, followed by the addition of ZrOCl_2_·8H_2_O (120 mg), TCPP-M (31 mg), and benzoic acid (1.20 g) mixed in DMF (8.0 ml). The mixture was sealed in a stainless autoclave with Teflon inner liner and heated at 120 °C for 24 h. After cooling down to ambient temperature, the crystals were harvested by centrifugation and washed with toluene for wiping off the dissociative C_60_ particles, and then with DMF, tetrahydrofuran (THF), and CH_2_Cl_2_ successively, for further cleaning the unreacted ligands and exchanging the solvent molecules. After drying in vacuum, CPCN-M (M = Fe, Co, or Ni) was gotten eventually. The recipe for PCN-M preparation is similar to that of CPCN-M, but without the participation of C_60_-toluene.

### Characterization methods

The scanning electron microscope (SEM) images were scanned on a Nova NanoSEM 450 microscope. The HREM images were observed on a JEM-2100F apparatus at an accelerating voltage of 200 kV. With Cu Kα at 40 kV and 40 mA, the XRPD patterns were tested with a Bruker D8 Advance diffractometer. With the KBr wafer, a Nicolet Nexus 470 spectrometer was used to record the FTIR spectra. The NMR spectroscopy was recorded with a Bruker AVANCE II 400M apparatus. The XPS was recorded on a Thermo Scientific Escalab 250Xi device with an Al Kα source. TG analyses in N_2_ atmosphere were performed with a TG209F1 apparatus. The UV-Vis absorption spectra, the luminescence emission spectra, and the luminescence decay profiles of the samples were recorded at ambient temperature with an FLS1000 from Edinburgh Instruments. After degassing the materials at 100 °C for 4 h, the N_2_ adsorption–desorption isotherms at 77 K were measured over a Micromeritics ASAP 2020 analyzer. *S*_BET_ was calculated at the *P*/*P*_0_ range of 0.05 to 0.15. The total pore volumes were calculated at the relative pressure of 0.95, and the nonlocal DFT (NLDFT) was used to estimate the pore size distribution.

### Static gas adsorption

Static adsorption experiments of CO_2_ (purity > 99.999%) and N_2_ (purity > 99.999%) over the sorbents were measured with the Micromeritics ASAP 2020 analyzer, respectively. The free space was determined using He (purity > 99.999%), with the assumption that He was not adsorbed. For pristine tests, the adsorption isotherms of CO_2_ and N_2_ were collected in a dark environment, with the sample cells immersed in an ice-water bath to keep the temperature at 0 °C or in a thermostatic water bath of 25 °C. For UV-Vis irradiation tests, a CEL-HXUV300 xenon lamp (optical power density: 2,000 mW cm^−2^; Beijing China Education AuLight Technology Co. Ltd.) was used with a VisREF optical filter to generate the exciting light at the wavelength of 350 to 780 nm. The xenon lamp was placed 20 cm away from the sample cells to provide excitation light source, and other operation conditions were similar to the pristine ones.

### Computational methods

The DFT and TDDFT calculations were performed by employing wB97XD functional implemented in Gaussian-16 package. The functional was proved to be robust for both DFT and TDDFT calculations [56,57]. With ultrafine numerical integration grids, self-consistent field procedures of full accuracy were performed with tight convergence and without any orbital symmetry constraints. The geometry relaxation was performed with the basis set of Def2-SVP. Four frontier excited states were involved in the TDDFT calculation with Tamm–Dancoff approximation in order to calculate the first excited state accurately.

## Data Availability

All data needed to evaluate the conclusions in the paper are present in the paper and/or the Supplementary Materials.
