# Supplementary material for: Photo-Responsive Carbon Capture over Metalloporphyrin-C60 Metal-Organic Frameworks via Charge-Transfer
Source: Research (Wash D C). 2023 Oct 24;6:0261. doi: 10.34133/research.0261 (PMC10595220; doi:10.34133/research.0261)
Supplement: Supplementary 1 — Figs. S1 to S18 Tables S1 to S3 [file research.0261.f1.pdf]

Supplementary Information

Photo-Responsive Carbon Capture over Metalloporphyrin-C_{60} Metal-Organic Frameworks via Charge-Transfer

Shi-Chao Qi, Zhen Sun, Zhi-Hui Yang, Yun-Jie Zhao, Jia-Xin Li, Xiao-Qin Liu, and Lin-Bing Sun*

State Key Laboratory of Materials-Oriented Chemical Engineering, Jiangsu National Synergetic Innovation Center for Advanced Materials (SICAM), College of Chemical Engineering, Nanjing Tech University, Nanjing, China, 211816.

*Address correspondence to: lbsun@njtech.edu.cn (L.B.S.)

The related definitions and formulas:

(1) Definition of *ESP*:

$$ESP\,(\boldsymbol{r}) = \Sigma_{\mathrm{N}}\,[Z_{\mathrm{N}}/|\boldsymbol{r}\text{-}\boldsymbol{R}_{\mathrm{N}}|] - \int[\boldsymbol{\rho}(\boldsymbol{r'})/|\boldsymbol{r} - \boldsymbol{r'}|]d\boldsymbol{r'}$$

in which Z_{N} and $\boldsymbol{R}_{\mathrm{N}}$ are the nuclear charge and its location, respectively, and $\boldsymbol{\rho}(\boldsymbol{r'})$ means the electrons density.

(2) Definition of electron-hole distribution:

$$\boldsymbol{\rho}_{\mathrm{hole}}(\boldsymbol{r}) = \sum_{\mathrm{i}\to\mathrm{m}} c_{\mathrm{i}\to\mathrm{m}}^2 \varphi_{\mathrm{i}}^2(\boldsymbol{r}) + \sum_{\mathrm{i}\to\mathrm{m}} \sum_{\mathrm{j}\to\mathrm{m}}^{\mathrm{i}\neq\mathrm{j}} c_{\mathrm{i}\to\mathrm{m}} c_{\mathrm{j}\to\mathrm{m}}\, \varphi_{\mathrm{i}}(\boldsymbol{r})\varphi_{\mathrm{j}}(\boldsymbol{r})$$

$$\boldsymbol{\rho}_{\mathrm{electron}}(\boldsymbol{r}) = \sum_{\mathrm{i}\to\mathrm{m}} c_{\mathrm{i}\to\mathrm{m}}^2 \varphi_{\mathrm{m}}^2(\boldsymbol{r}) + \sum_{\mathrm{i}\to\mathrm{m}} \sum_{\mathrm{i}\to\mathrm{n}}^{\mathrm{m}\neq\mathrm{n}} c_{\mathrm{i}\to\mathrm{m}} c_{\mathrm{i}\to\mathrm{n}}\, \varphi_{\mathrm{m}}(\boldsymbol{r})\varphi_{\mathrm{n}}(\boldsymbol{r})$$

in which φ_{i} (φ_{j}) and φ_{m} (φ_{n}) represent the originally occupied and virtual molecular orbitals, respectively, and c is the configuration coefficient.

(3) Definition of electron-hole delocalization index (EDI & HDI):

$$EDI = 100\sqrt{\int \boldsymbol{\rho}_{\mathrm{electron}}^2\,(\boldsymbol{r})d\boldsymbol{r}}\ ;\ HDI = 100\sqrt{\int \boldsymbol{\rho}_{\mathrm{hole}}^2\,(\boldsymbol{r})d\boldsymbol{r}}$$

(4) Variation of μ at c-axis with respect to ground state ($\Delta\mu$):

$$\Delta\mu = \iint c\boldsymbol{\rho}_{\mathrm{hole}}(\boldsymbol{r})dcd\boldsymbol{r} - \iint c\boldsymbol{\rho}_{\mathrm{electron}}(\boldsymbol{r})dcd\boldsymbol{r}$$

in which c means the coordinates at c-axis direction.

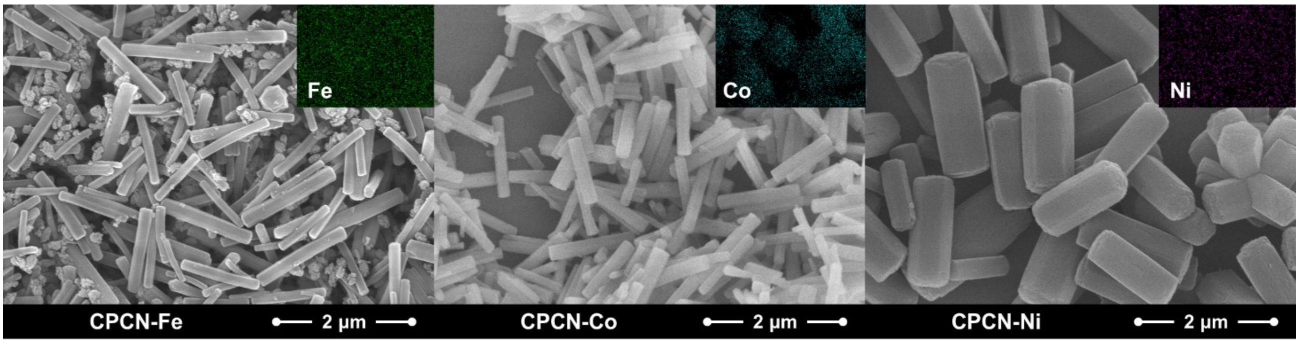

Fig. S1. The SEM images of CPCN-M samples.

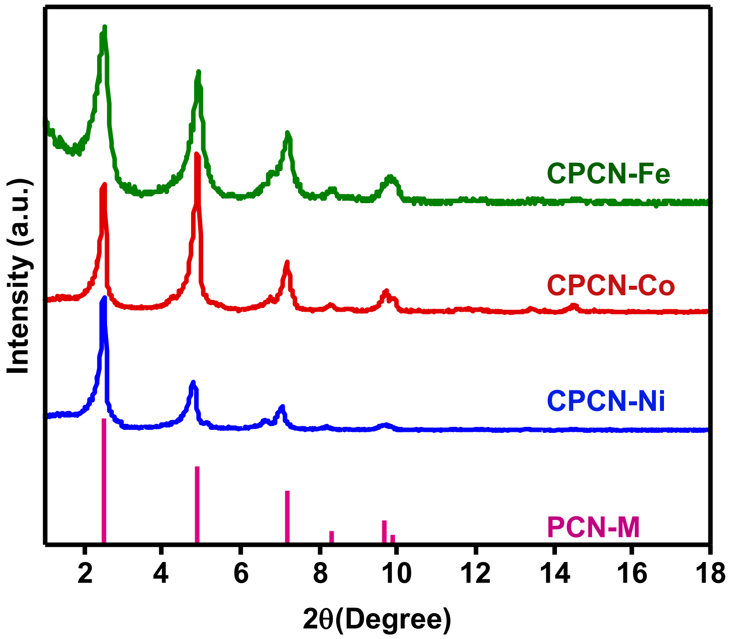

Fig. S2. The XRPD patterns of the CPCN-Ms and the pair distribution function of the PCN-M reported.

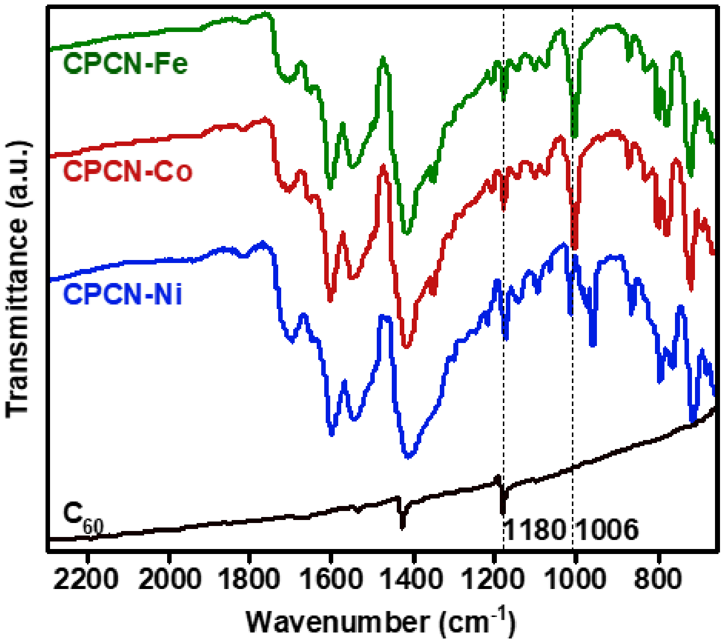

Fig. S3. The FTIR spectra of CPCN-Ms and C_{60}.

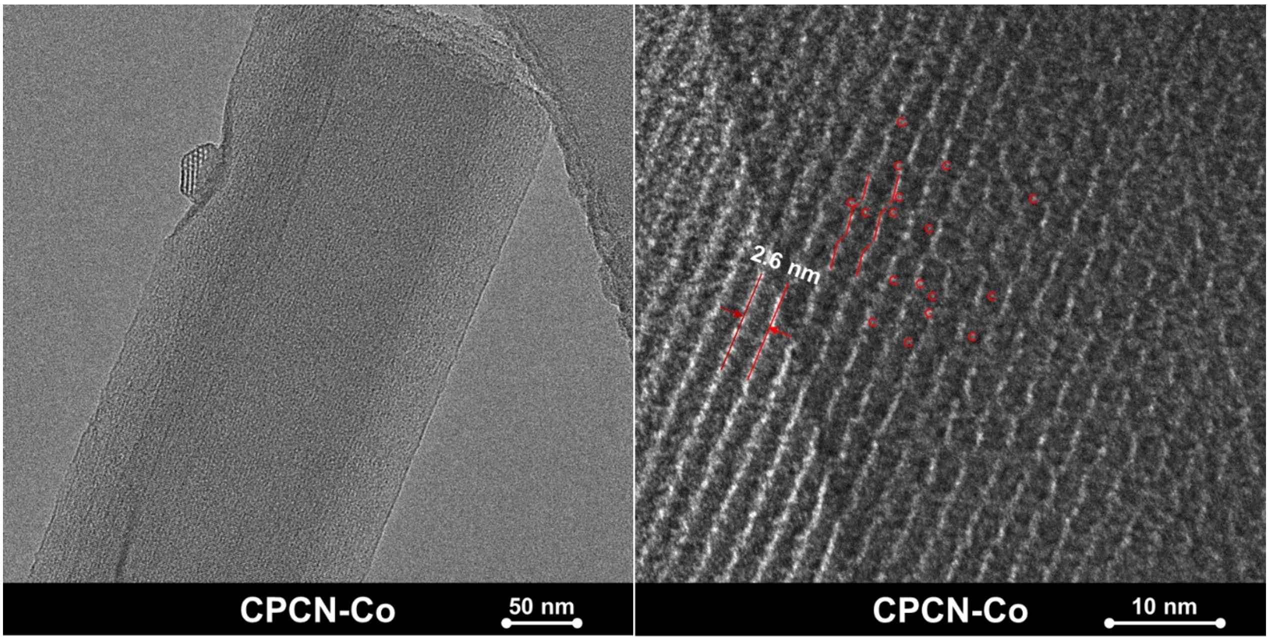

Fig. S4. The HREM images of the representative CPCN-Co. The red broken lines indicate the distorted lattice, and the red cycles indicate the doped C_{60}.

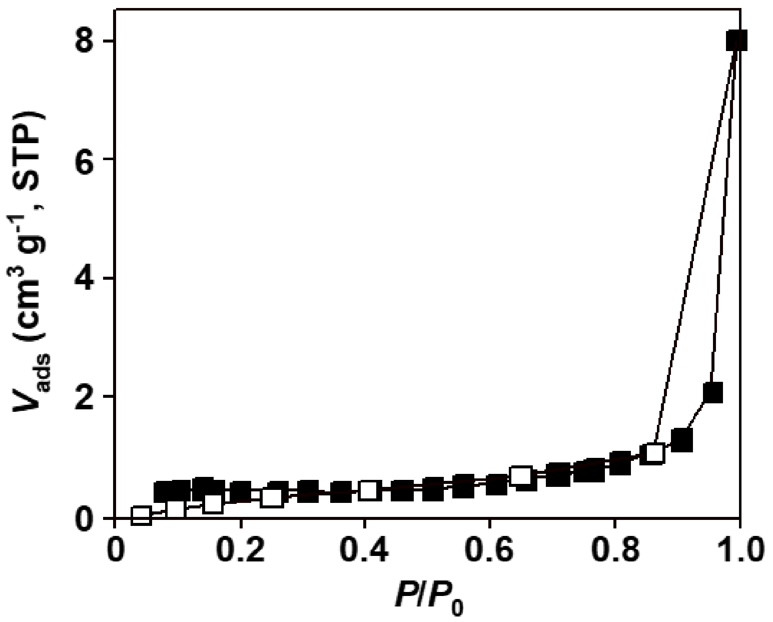

Fig. S5. The N_2 adsorption-desorption isotherm of the C_{60}.

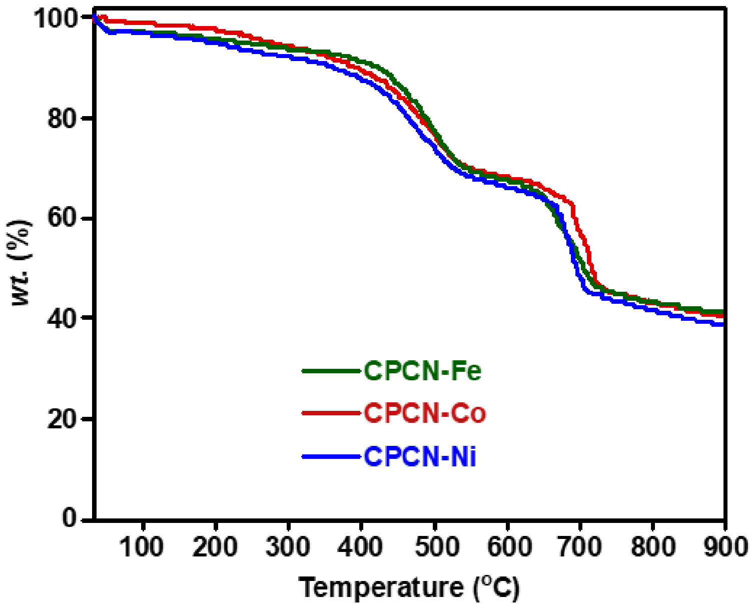

Fig. S6. The TG profiles of CPCN-M samples.

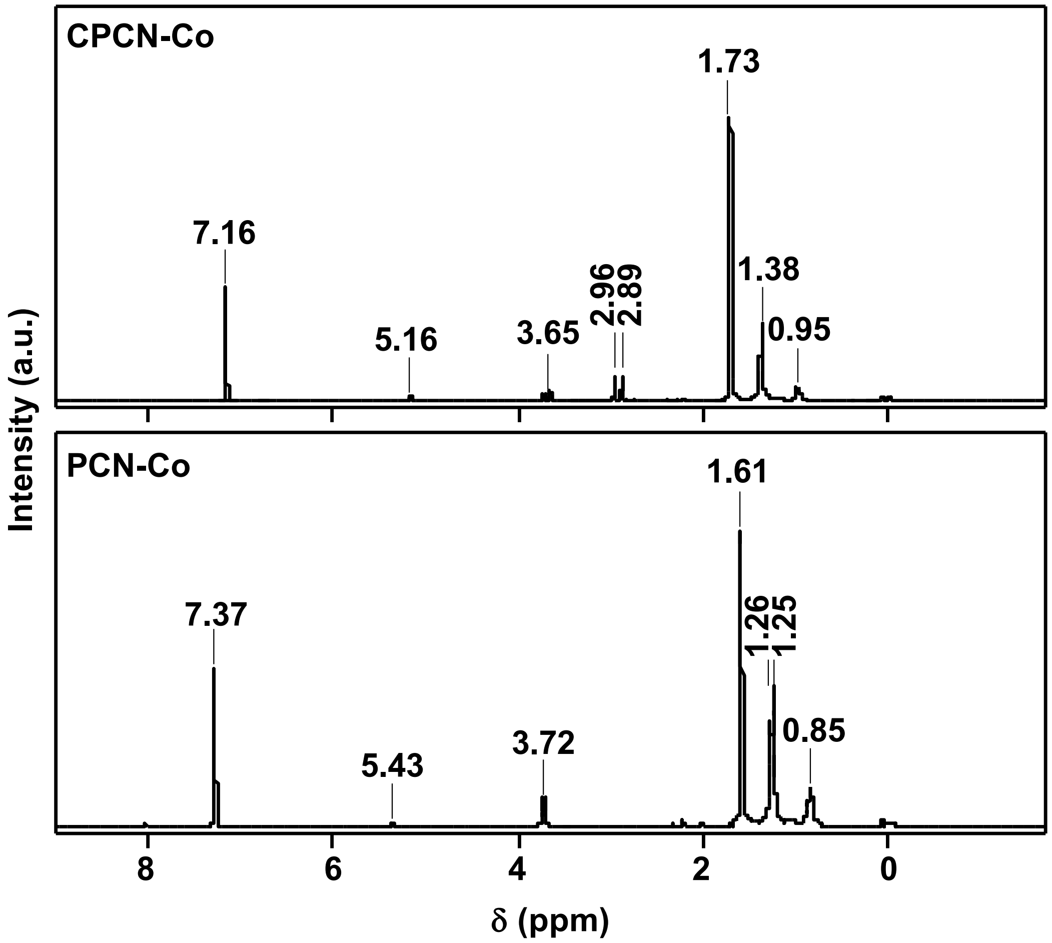

Fig. S7. The H-NMR spectra of the representative CPCN-Co and its contrast PCN-Co. CPCN-Co (C_2D_6SO, 400 MHz): *δ* 2.89, 2.96, 3.65, 5.16, 7.16 (s, 8H, porphin ring-*H*), *δ* 1.73 (s, 8H, proximal phenyl-*H*), *δ* 0.95-1.38 (m, 8H, distal phenyl-*H*). PCN-Co (C_2D_6SO, 400 MHz): *δ* 3.72, 5.43, 7.37 (s, 8H, porphin ring-*H*), *δ* 1.61 (s, 8H, proximal phenyl-*H*), *δ* 0.85-1.26 (m, 8H, distal phenyl-*H*).

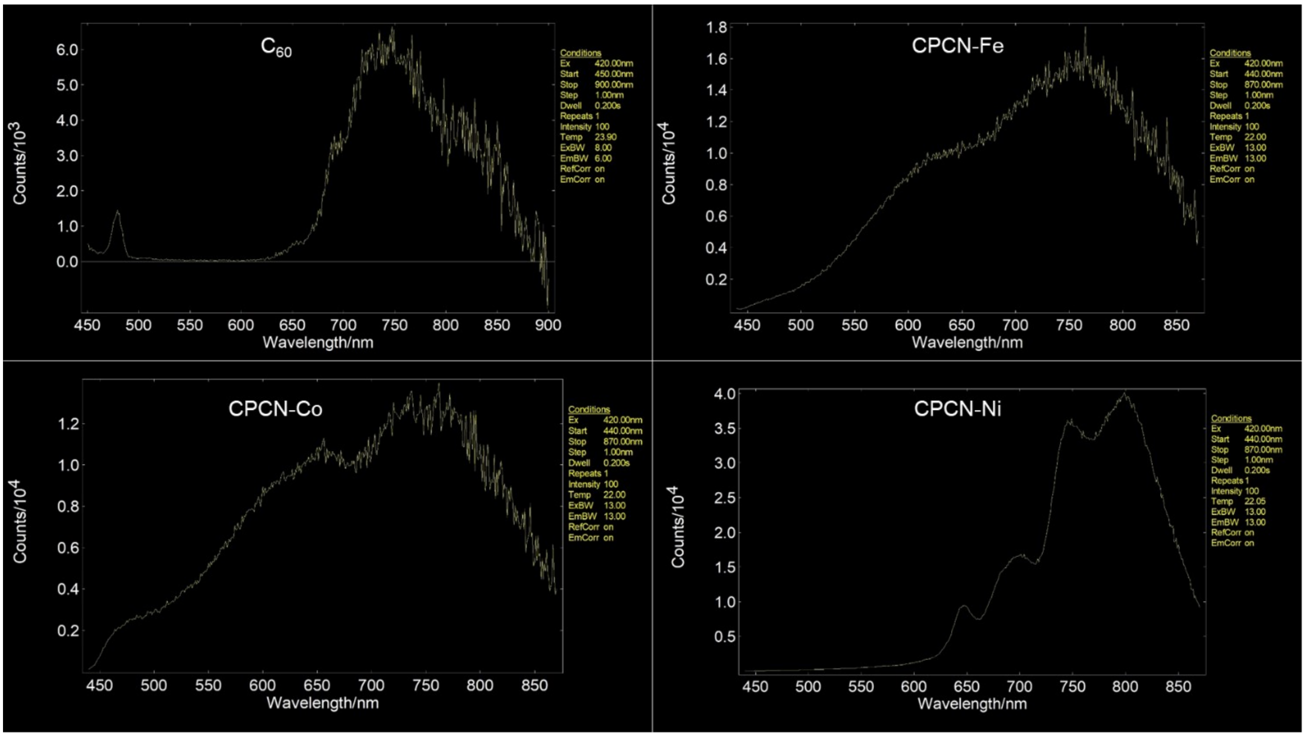

Fig. S8. The phosphorescent radiation spectra of solid samples excited with the Vis = 420 nm at ambient temperature.

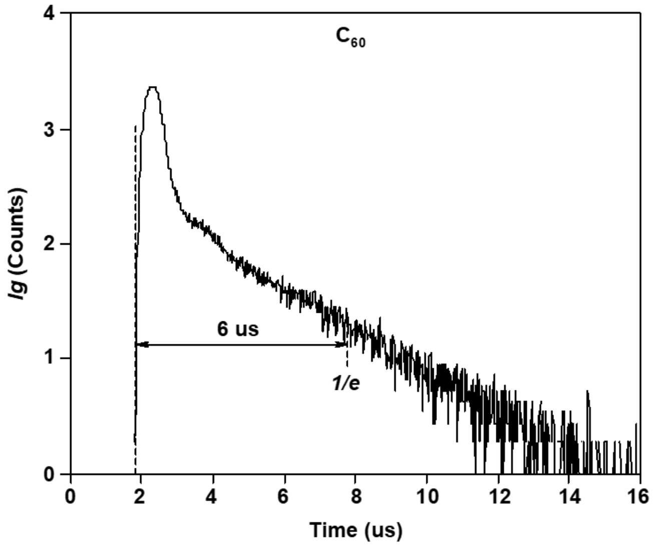

Fig. S9. The phosphor decay profile of the C_{60} at 750 nm, in which the *1/e* indicates the effective phosphorescence lifetime.

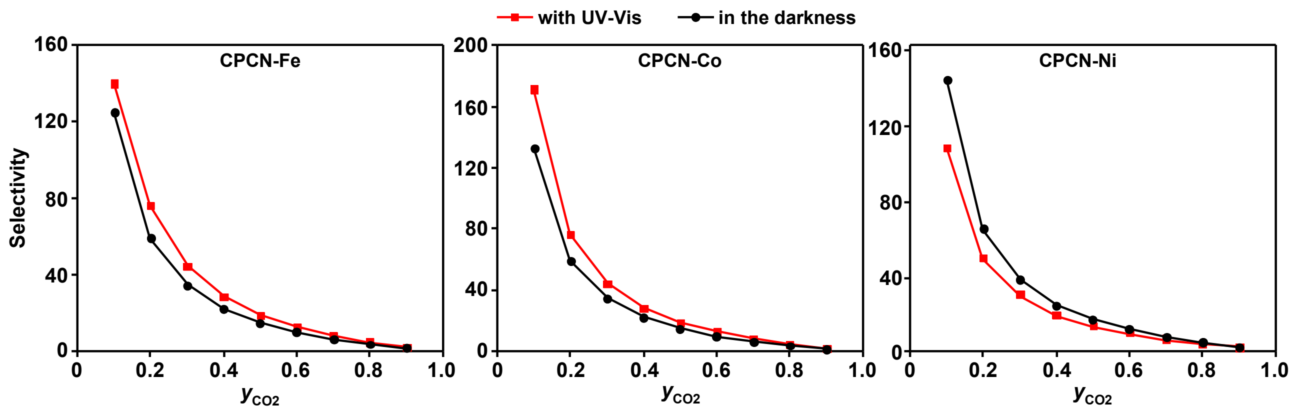

Fig. S10. The IAST selectivity of CO_2 towards N_2 at 0 °C and 1 bar.

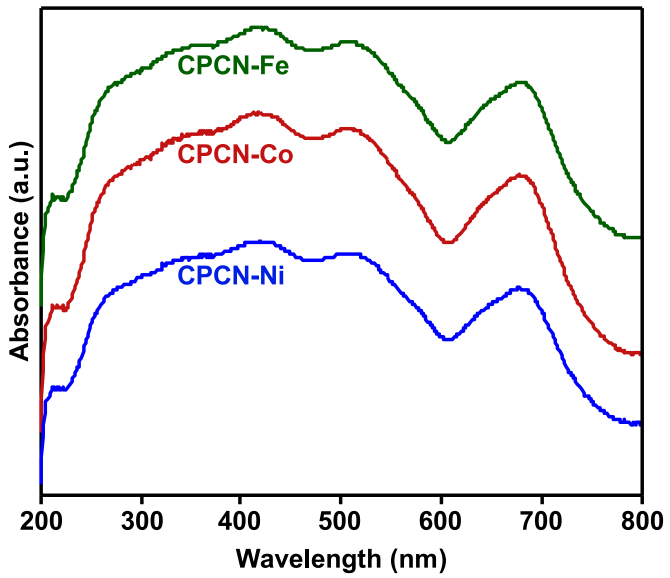

Fig. S11. The UV-Vis absorption spectra of solid samples.

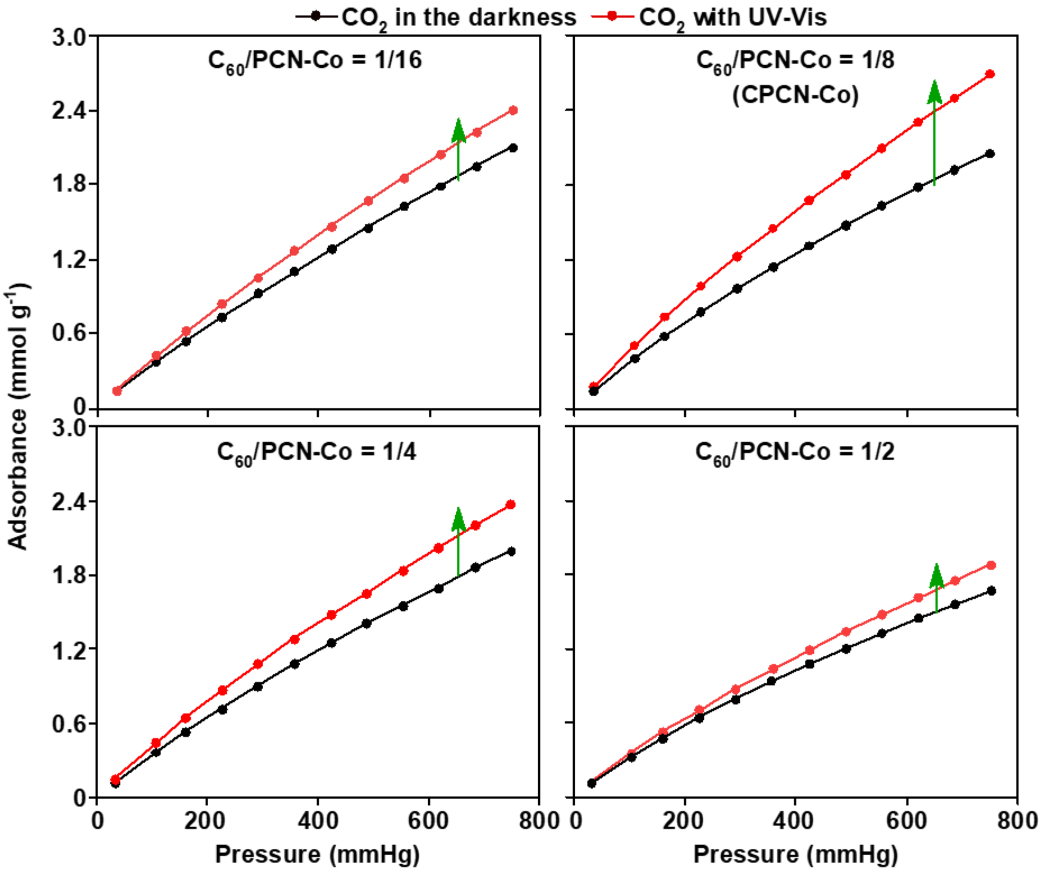

Fig. S12. The static adsorption isotherms of CO_2 tested with UV-Vis irradiation and in the darkness over the CPCN-Co samples with different mass ratios of C_{60}/PCN-Co at 0 °C, in which the green arrows indicate the variation trend of the UV-Vis CO_2 adsorption isotherm with respect to that in the darkness.

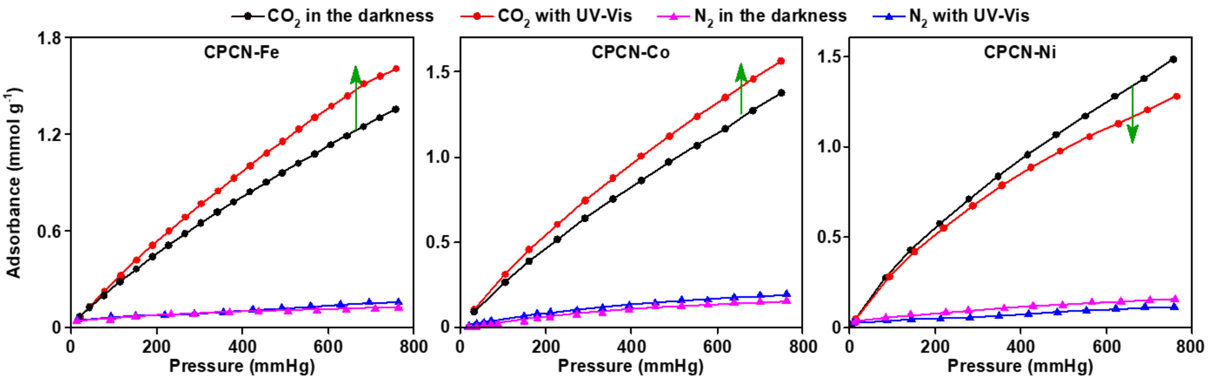

Fig. S13. The static adsorption isotherms of CO_2 and N_2 tested with UV-Vis irradiation and in the darkness over the CPCN-Ms at 25 °C, in which the green arrows indicate the variation trend of the UV-Vis CO_2 adsorption isotherm with respect to that in the darkness.

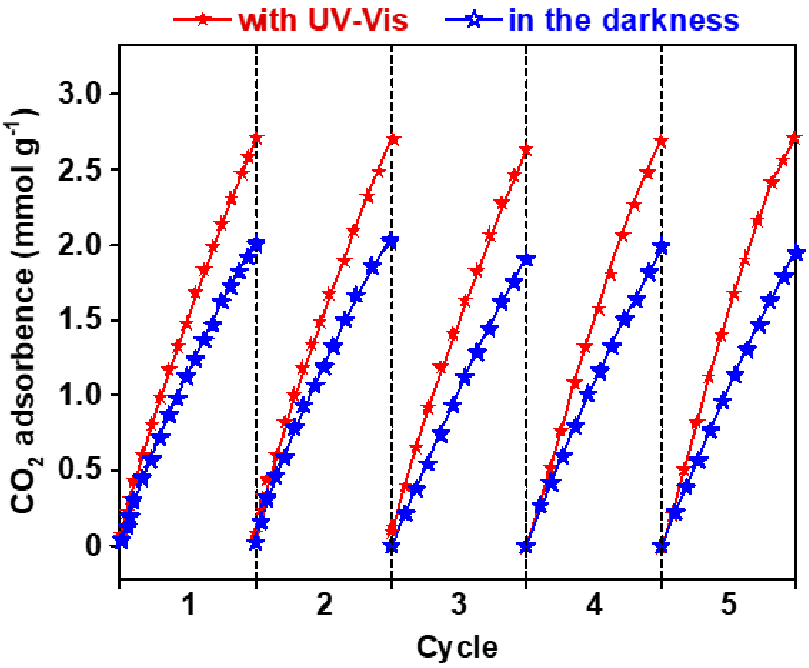

Fig. S14. The recyclability test for the representative CPCN-Co.

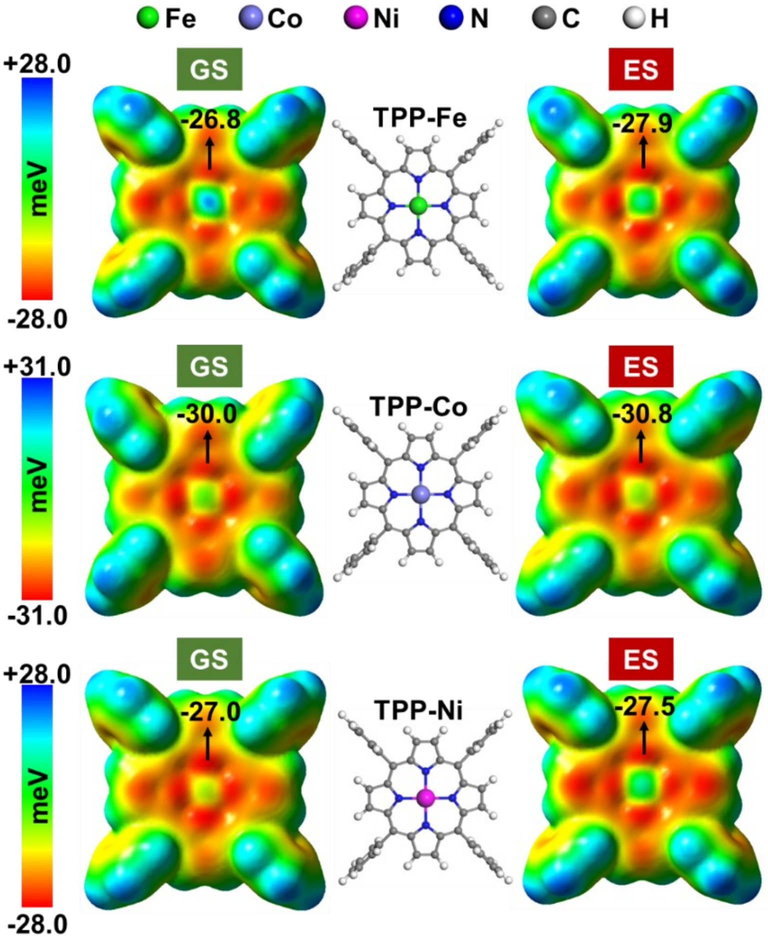

Fig. S15. The molecular surface ESPs of the TPP-M with the density isovalue = 1×10^{-3} at GS and at ES.

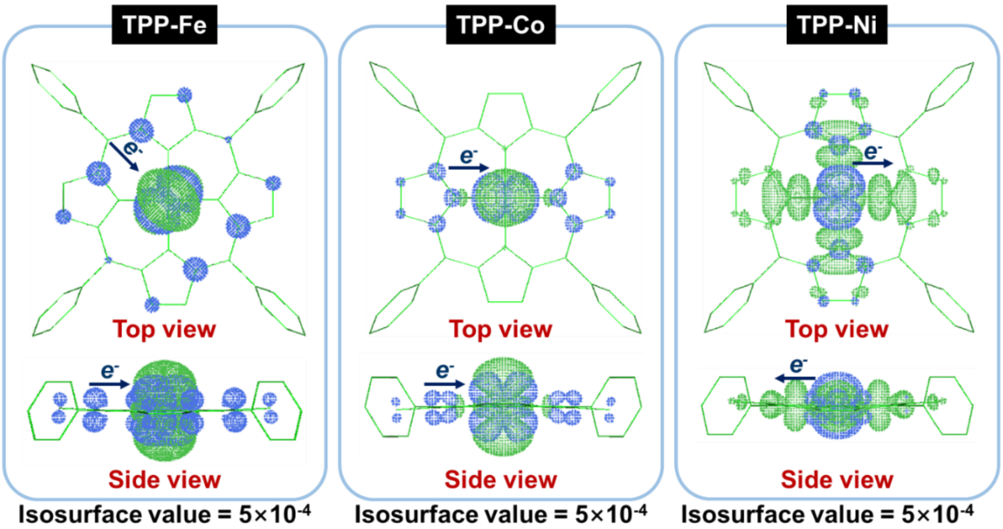

Fig. S16. The electron-hole distribution at ES of the TPP-M (green area: electrons distribution; blue area: holes distribution).

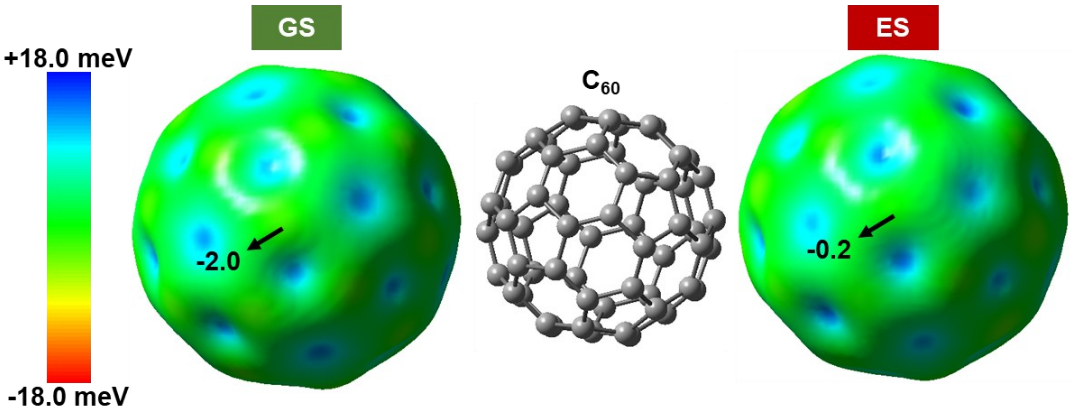

Fig. S17. The molecular surface ESPs of C_{60} with the density isovalue = 1×10^{-3} at GS and at ES.

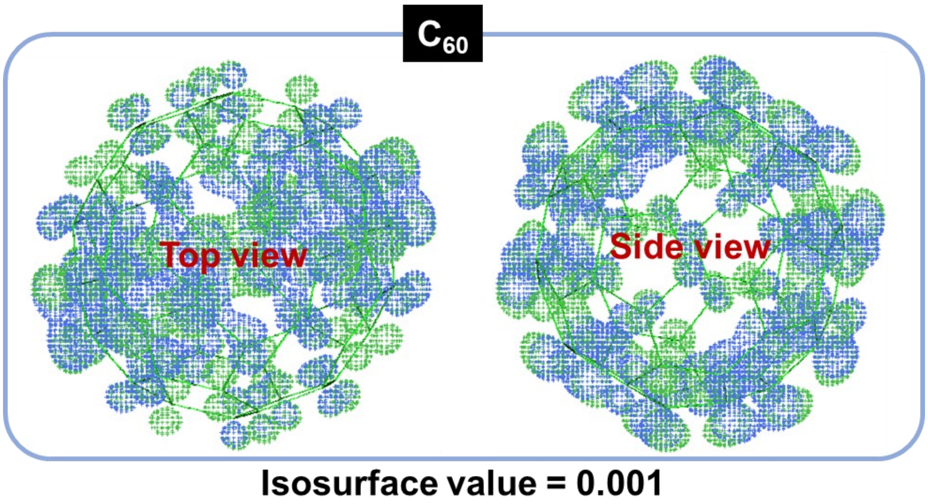

Fig. S18. The electron-hole distribution at ES of C_{60} (green area: electrons distribution; blue area: holes distribution).

Table S1. The textural properties of CPCN-Ms and the gas adsorption uptakes at 0 °C and 1 bar.

Sorbent	S_{BET}	V_{pore}	CO_2 uptake (mmol g^{-1})		N_2 uptake (mmol g^{-1})	
	m^2 g^{-1}	cm^3 g^{-1}	Pristine	UV-Vis	Pristine	UV-Vis
CPCN-Fe	2100	2.10	2.08	2.50	0.20	0.23
CPCN-Co	2160	2.15	2.05	2.69	0.14	0.20
CPCN-Ni	2130	2.06	2.43	1.99	0.13	0.12

Table S2. The CO_2 adsorption performances and the deforming units of some representative photo-responsive sorbents during the past decade.

Sorbent	Deforming unit	S_{BET} ($m^2\ g^{-1}$)	CO_2 adsorption			Ref.
			Uptake (mmol g^{-1})	*R.C.*[a]	T, P	
PCN-123	2-(Phenyldiazenyl)terephthalate	--	1.02 (no light) / 0.75 (UV)	-26%	22°C, 1 bar	*19*
ECUT-15	4,4'-Diazene-1,2-diyldibenzoate acid	--	0.26 (no light) / 0.14 (UV)	-46%	25 °C, 1 bar	*20*
$Zn(AzDC)(4,4'\text{-}BPE)_{0.5}$	4,4'-Dicarboxylate	126	1.23(no light) / 0.53 (UV-Vis)	-57%	30 °C, 1 bar	*45*
azo-IRMOF-10	2-Azobenzene-4,4'-biphenyldicarboxylate	4086	0.71 (no light) / 0.65 (UV)	-8%	25 °C, 1 bar	*46*
Azo-DMOF-1	2-(Phenyldiazenyl)terephthalate	581	3.26 (no light) / 1.61 (UV)	-51%	0 °C, 1 bar	*47*
A_2P_2@MS	4-(3-Triethoxysilylpropyl-ureido)azobenzene	797	2.55 (Vis) / 1.65 (UV)	-35%	0 °C, 1 bar	*48*
Azo-COP-2	Cross-coupling 1,2-di-*p*-tolyldiazene	554	2.12 (no light) / 1.66 (UV)	-22%	0 °C, 1 bar	*49*
P_3/azoMOF	Grafted azobenzene	324	2.94 (Vis) / 2.21 (UV)	-25%	25 °C, 1 bar	*50*
SP/CTA^+/Mont	Spiropyran	~1	0.43 (no light) / 0 (UV)	-100%	27 °C, 1 bar	*51*
HCPs@Azo	Azobenzene	984	1.68 (Vis) / 2.54 (UV)	+51%	0 °C, 1 bar	*52*
CPCN-Co	Nondeforming	2160	2.05 (no light) / 2.69 (UV-Vis)	+31%	0 °C, 1 bar	This work
CPCN-Fe	Nondeforming	2100	1.32 (no light) / 1.58 (UV-Vis)	+20%	25 °C, 1 bar	This work

a: *R.C.*, rate of change.

Table S3. The indexes of the excited states for the adsorption sites.

Index	TPP-Fe	CTPP-Fe	TPP-Co	CTPP-Co	TPP-Ni	CTPP-Ni
Hole contribution from the -M/%	92.4	88.8	94.7	94.2	94.9	98.2
Electron contribution from the -M/%	101.2	91.4	100.6	95.9	71.3	70.8
Difference between electron and hole/%	+8.8	+2.6	+5.9	+1.7	-23.6	-27.4
Hole delocalization index/a.u.	39.4	33.4	45.0	44.1	49.5	46.6
Electron delocalization index/a.u.	36.4	32.1	40.8	39.7	42.0	41.9
Variation of μ at *c*-axis with respect to ground state/$\times 10^{-4}$ a.u.	1.7	970.7	23.0	737.3	13.6	673.6
